# Identification and validation of palmitoylation-related signature genes based on machine learning for prostate cancer

**DOI:** 10.1371/journal.pone.0338407

**Published:** 2025-12-04

**Authors:** Qijun Wo, Jiafeng Shou, Jun Shi, Lei Shi, YunKai Yang, Yifan Wang, Liping Xie

**Affiliations:** 1 Department of Urology, First Affiliated Hospital, School of Medicine, Zhejiang University, Hangzhou, China; 2 Urology & Nephrology Center, Department of Urology, Zhejiang Provincial People’s Hospital, Hangzhou, Zhejiang, China; 3 Department of Urology, The Second People’s Hospital of Fuyang, Hangzhou, Zhejiang, China; 4 Cancer Center, Department of Radiation Oncology, Zhejiang Provincial People’s Hospital (Affiliated People’s Hospital), Hangzhou Medical College, Hangzhou, Zhejiang, China; University of Helsinki: Helsingin Yliopisto, FINLAND

## Abstract

Prostate cancer (PCa) remains a leading cause of cancer-related mortality in men, with challenges in diagnosis and treatment due to tumor heterogeneity. This study identifies palmitoylation-related signature genes as potential diagnostic and therapeutic targets. Integrating GEO datasets, six differentially expressed genes (DEGs) linked to palmitoylation were identified. Machine learning algorithms (LASSO, RF, SVM) selected three core genes: TRPM4, LAMB3, and APOE. A diagnostic model based on these genes achieved an AUC of 0.929, demonstrating robust accuracy in distinguishing PCa from normal tissues. Functional analysis revealed roles in lipid metabolism and immune modulation, with ssGSEA highlighting correlations between key genes and immune cell infiltration. Experimental validation showed that LAMB3 overexpression suppressed PCa cell proliferation, migration, and invasion, while knockdown enhanced these processes. Molecular docking identified diethylstilbestrol as a potential therapeutic agent targeting LAMB3 and APOE. These findings emphasize the clinical relevance of palmitoylation-related genes in PCa diagnosis and therapy, offering novel biomarkers and insights for personalized treatment strategies.

## 1. Introduction

Prostate cancer (PCa) ranks among the most prevalent malignant tumors in males worldwide [1,2]. According to global cancer statistics, PCa represents the second leading cause of cancer-related mortality in the male population worldwide. The diagnosis of prostate cancer currently relies on a combination of serum prostate-specific antigen (PSA) testing, digital rectal examination (DRE), imaging modalities (e.g., multiparametric MRI), and histopathological confirmation via prostate biopsy [3]. Despite their critical role in early detection, these approaches are limited by the marked heterogeneity of prostate cancer, which contributes to persistent challenges in overdiagnosis and overtreatment [4]. Therapeutic strategies encompass conventional modalities such as surgical resection, radiotherapy, androgen deprivation therapy (ADT), and chemotherapy, as well as emerging strategies including molecularly targeted therapies and immunotherapies [5]. However, the development of castration-resistant prostate cancer (CRPC), characterized by resistance to androgen deprivation, remains a major therapeutic hurdle. This resistance underscores the urgent need for innovative approaches to overcome treatment-refractory disease and improve clinical outcomes [6,7].

Protein acylation (including S-prenylation, N-myristoylation, and S-palmitoylation), as a class of dynamic and reversible post-translational modifications (PTMs), plays a central role in cell signal transduction and sub – organelle function regulation by modulating protein membrane localization, stability, and interaction networks [8,9]. Among them, S – palmitoylation modifies proteins by covalently attaching to the sulfhydryl group of cysteine residues through a thioester bond and is directly involved in protein membrane anchoring, conformational maturation, and vesicle transport. This modification widely influences physiological processes such as neurotransmission and immune response by dynamically regulating the activities of membrane receptors (e.g., GPCRs), ion channels (e.g., the TRP family), and cell adhesion molecules (e.g., integrins) [9–12].

In recent years, studies have found that palmitoylation enhances the nuclear translocation ability and transcriptional activity of the androgen receptor, promoting the proliferation of prostate cancer cells [13–15]. It also remodels the lipid metabolism pathway in the prostate cancer micro – environment by modifying key metabolic proteins such as UQCRC2 and N0DUFS1 [16,17]. Therefore, a comprehensive analysis of the key regulatory genes of palmitoylation is necessary and has become an important breakthrough point for the precision treatment of prostate cancer.

This study aims to identify and validate palmitoylation-related gene signatures associated with prostate cancer using the Gene Expression Omnibus (GEO) database and machine learning approaches. The LASSO regression, random forest (RF), and support vector machine (SVM) algorithms were employed to construct a diagnostic model for selecting key genes with diagnostic value. The immune infiltration characteristics of these key genes were analyzed through single-sample gene set enrichment analysis (ssGSEA), and a molecular interaction regulatory network was established using Cytoscape software. Subsequently, molecular docking techniques were applied to predict potential therapeutic agents targeting core genes. Finally, the expression of critical genes was validated via quantitative real-time PCR (qRT-PCR) and Western blot (WB) experiments to elucidate their complex mechanisms in tumor regulation and diagnostic significance in prostate cancer (PCa).

## 2. Methods and materials

### 2.1. Data acquisition

**Fig 1:**
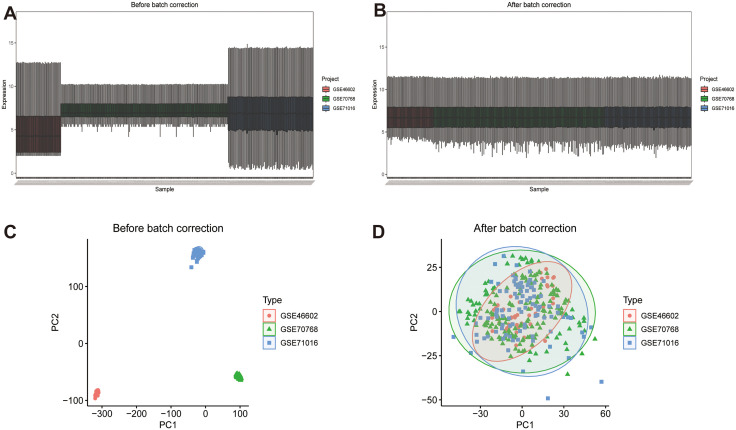
Dataset merge and calibration. **(A)** Box plot of the integrated dataset before calibration **(B)** and after calibration. **(C)** PCA map of the integrated dataset before calibration and **(D)** after calibration. PCA: principal component analysis.

We utilized the Gene Expression Omnibus (GEO) database to collect information on PCa. In our research, the datasets GSE46602, GSE70768, and GSE71016 were extracted as the training sets, while GSE69223 was used as the test set. Initially, the datasets GSE46602, GSE70768, and GSE71016 were subjected to batch effect correction using the “sva” R package. To ensure consistency and remove any potential biases, the datasets were combined, and principal component analysis (PCA) was used to simplify the data and eliminate batch effects (Fig 1A-D). The datasets included in this study were 197 prostate adenocarcinoma samples and 134 adjacent normal prostate tissues. All cancer samples were treatment-naïve, localized PCa cases (no metastatic or CRPC samples were included). Normal samples were derived from histologically confirmed non-cancerous regions of prostates from age-matched donors (median age: 62 years). Gleason scores for cancer samples were distributed as follows: Gleason = 4–6 (n = 47), Gleason = 7 (n = 132), Gleason = 8–10 (n = 18). Subsequently, differential expression analysis was performed on the integrated dataset using the “limma” R package to identify differentially expressed genes (DEGs) meeting the criteria of |logFC| > 1 and p.adjust < 0.05. Palmitoylation-related genes were obtained from the GeneCards database (https://www.genecards.org/) using “palmitoylation” as the search keyword, a total of 1,611 genes were obtained. The filtering criteria of “Protein Coding” and a relevance score > 2 resulted in the identification of 1306 related genes (S1 Table). Venn diagrams were used to identify DEGs associated with palmitoylation. Heatmaps were also generated to illustrate the expression patterns of palmitoylation-related DEGs.

### 2.2. Enrichment analysis

Gene Ontology (GO) analysis was conducted to perform large-scale functional enrichment, considering biological processes (BP), molecular functions (MF), and cellular components (CC). The “limma,” “clusterProfiler,” “enrichplot,” and “org.Hs.e.g.,db” R packages were used for enrichment analysis and visualization of palmitoylation-related DEGs. In Gene Set Enrichment Analysis (GSEA), genes were compared between two biological states to determine statistical differences. Based on logFC permutations of GEO data, GSEA was used to investigate differences in biological processes between groups. The GSVA package was used to calculate enrichment scores for important pathways in the MSigDB database by analyzing the gene expression data from each sample.

### 2.3. Screening characteristic-related biomarkers via machine learning

Next, logistic regression analysis was performed on the DEGs and their expression matrices in the integrated dataset. Three machine learning methods were employed to evaluate key feature genes in PCa. The Least Absolute Shrinkage and Selection Operator (LASSO) algorithm and the “glmnet” package were used for dimensionality reduction and feature selection between PCa patients and normal samples. Determine the λ value with the smallest error through 10-fold cross-validation. The Random Forest (RF) algorithm was applied to select feature genes by modeling multiple decision trees through ensemble learning, counting the prediction results of each tree, and selecting the optimized outcome. By drawing the OOB error curve, the minimum number of trees when the error is stable is selected to ensure the stability of the model, and then the genes with higher importance rankings are screened. Additionally, Support Vector Machine Recursive Feature Elimination (SVM-RFE) was used to assess the average error rate via 10-fold cross-validation to identify the point with the least error. LASSO regression was chosen for high-dimensional data shrinkage and collinearity reduction, RF for non-linear feature importance ranking via ensemble decision trees, and SVM-RFE for recursive elimination of low-weight features. Integrating these methods balances LASSO’s sparsity, RF’s robustness to noise, and SVM’s margin maximization, reducing overfitting risk. Venn diagrams were then used to identify overlapping genes from the three algorithms.

### 2.4. Diagnostic value of the biomarkers in PCa

The “ggpubr” R package was used to examine the differences in these hub genes and evaluate the predictive value of the established biomarkers. A significance level of P < 0.05 was considered statistically significant. Subsequently, the “pROC” R package was used to generate ROC curves in a training group consisting of 197 PCa cases and 134 normal controls. The area under the curve (AUC) values were calculated to assess the diagnostic impact of palmitoylation-related signature genes in PCa compared to normal samples. The “rms” R package was employed to construct nomograms, and Decision Curve Analysis (DCA) was introduced using the “ggDCA” package to determine model accuracy. DCA evaluated whether the clinical prediction model had an optimal curve direction. Additional calibration analysis was performed to plot curves reflecting the diagnostic model’s capability based on palmitoylation-related signature genes.

Considering the inherent “black box” nature of machine learning models, we implemented the SHapley Additive exPlanations (SHAP) algorithm to quantify the contribution of each feature to the prediction result. This method assigns a SHAP value to each feature, thereby enabling an interpretable assessment of the impact of that feature on the model’s prediction.

### 2.5. Analysis of correlations between identified genes and immune cell infiltration

Single-sample Gene Set Enrichment Analysis (ssGSEA) was used to classify 28 different types of immune cell matrices. A reference set of 28 immune cell subtypes was used to estimate the presumed abundance of immune cells. Immune cell matrix infiltration was generated based on a significance level of P < 0.05. The “corrplot” program was used to illustrate the associations between core genes and the infiltration of 28 different immune cells, establishing correlations in heatmaps and boxplots.

### 2.6. mRNA-RBP, mRNA-TF, and mRNA-drug interaction networks

The StarBase v3.0 database was used to predict target RNA-binding proteins (RBPs) for 46 key genes, resulting in an mRNA-RBP regulatory network. Interaction networks of genes with similar functions were predicted using key genes retrieved from the STRING database (http://string-db.org/). Additionally, the Drug Signatures Database (DSigDB) (https://dsigdb.tanlab.org/) was used to predict potential drugs interacting with key genes. Cytoscape software was employed to generate drug regulatory and interaction networks.

### 2.7. Molecular docking

Based on the results of the drug enrichment analysis, laminin subunit beta 3 (LAMB3) and apolipoprotein E (APOE) were selected as targets, with diethylstilbestrol identified as a potential drug. The drug structure was obtained from the PubChem database (https://pubchem.ncbi.nlm.nih.gov/). We used the CBDOCK online site (https://cadd.labshare.cn/) to predict and visualise the protein structures of core genes.

### 2.8. Cell culture, transfection and cohorts establish

The human PCa cell lines, LNCaP and PC3, were routinely maintained in RPMI-1640 medium (Gibco, USA) supplemented with 10% fetal bovine serum (FBS, HyClone, USA) and 1% penicillin/streptomycin (Thermo Fisher Scientific) under standard culture conditions (37°C, 5% CO2). LNCaP and PC3 cells were evenly plated in 6-well plates. Once the two cell lines reached approximately 80–90% confluence, they were transfected following the provided instructions. Due to the presence of the puromycin resistance gene in the lentiviral plasmid, lentivirus-infected cells can be selected by puromycin. Eventually, two comparison cohorts were formed (OE-Ctrl, OE-LAMB3; Sh-Ctrl, Sh-LAMB3).

### 2.9. RT-qPCR analysis

RNA was extracted from PCa cells using TriZol (R0016, Beyotime, China). The extracted RNA was reverse-transcribed into complementary DNA (cDNA) using the PrimeScript™ cDNA Synthesis Kit (6210A, Takara, Japan). Quantified expressions were detected using SYBR Green qPCR Master Mix and the 2-ΔΔCq method.

The RT‑PCR primer sequences (Invitrogen, CA, USA) were LAMB3 (forward) 5′‑CCAAAGGTGCGACTGCAATG‑3′ and (reverse) 5′‑AGTTCTTGCCTTCGGTGTGG‑3′. GAPDH (forward) 5′‑ACAACTTTGGTATCGTGGAAGG‑3′ and (reverse) 5′‑GCCATCACGCCACAGTTTC‑3′.

### 2.10. Western blot analysis

Proteins were separated by electrophoresis on a 4%−12% SDS-PAGE gel and transferred to a PVDF membranes, which were blocked with a 5% skim milk solution at room temperature for 1 hour. Subsequently, the membranes were incubated overnight at 4°C with primary antibodies against LAMB3 Rabbit Polyclonal Antibody (HA500480; 1:1000; HUABIO; China) and β-actin (R1207-1; 1:10,000; HUABIO; China). Following that the membranes were incubated with secondary antibodies (5151P; 1:3,000; CST; China) was performed at room temperature for 1 hour. The membranes were visualized using the Odyssey DLx Imaging System (LICORbio™, USA).

### 2.11. Cell proliferation assay

#### 2.11.1. Cell counting Kit-8.

Sh-Ctrl, Sh-LAMB3, OE-Ctrl, and OE-LAMB3 cell lines of LNCaP and PC3 were plated in 96-well plates with 4 replicate wells of 5000 cells/well per cell line. The plates were incubated at 37°C in a 5% CO_2_ atmosphere for continuous monitoring over 5 days. The CCK-8 Reagent Assay Kit (C0037, Beyotime, China) was added at 10 µl per well. PCa cells were further incubated at 37°C in a 5% CO_2_ for 1 hour, andthe optical density (OD) was measured using a microplate reader at a wavelength of 450 nm.

#### 2.11.2. EdU.

Cell proliferation experiments were conducted using the BeyoClick™ EdU Cell Proliferation Kit (C0078S, Beyotime, China). First, Click reaction (50 μl) containing EdU (20 μM) was prepared and added to a 96-well plates (5000 cells/well), followed by incubation for 2 hours. Cells were fixed with 4% paraformaldehyde for 30 minutes. Subsequently, 0.5% Triton X-100 was added for cell permeabilization. After discarding the waste liquid, Azide Alexa Fluor 594 (a fluorescent-labeled small molecule azide probe) and Hoechst 33342 staining solution were added successively and incubated in the dark for 30 minutes. Finally, the staining solution was discarded and proliferating cells were observed under a fluorescence microscope (EVOS™ M7000 imaging system, thermoFisher, USA) and an observation objective (Olympus™ 20X); Finally, ImageJ software was used to calculate the EdU-positive cells in the field of view.

### 2.12. Colony formation assay

Cultured cells in the logarithmic growth phase were diluted to 5000 cells per well and seeded into 6-well plates which containing culture medium. The cells were cultured at 37°C in a 5% CO_2_ for 2 weeks. Afterward, the cells were washed twice with PBS and fixed with 4% paraformaldehyde for 30 minutes. Colonies were stained with 0.1% crystal violet for 15 minutes and washed with water. Finally, clones consisting of more than 10 cells were counted.

### 2.13. Transwell migration/invasion assay

#### 2.13.1. Transwell migration assay.

An 8 µm Transwell chamber (3395, Corning, USA) was placed into a 24-well plate. RPMI-1640 medium containing 20% FBS was added to the lower chamber, and a cell suspension in basal medium was added to the upper chamber, with a cell density of 5000 cells/well for each stable cell line. After incubation at 37°C for 18 hours, the Transwell chamber was removed and washed with PBS. Cells that had not migrated were wiped from the upper surface of the chamber using a cotton swab. Methanol was added to fix the cells for 20 minutes. Finally, 0.01% crystal violet solution was added for 15 minutes. Three random fields were observed under an inverted microscope for imaging and recording.

#### 2.13.2. Transwell invasion assay.

Compared to the Transwell migration assay, Matrigel (354230, Corning, USA) was applied to the bottom of the chamber before seeding tumor cells. Tumor cells degraded from the Matrigel by secreting hydrolytic enzymes and then passed through the pores to the lower surface of the chamber. This experiment simulates the process of tumor cells hydrolyzing the extracellular matrix in vitro to accelerate invasion and metastasis. The remaining steps were consistent with the migration assay.

#### 2.13.3. Wound healing assay.

PC3 and LNCaP cells were seeded into 6-well plates and cultured until they reached 95% confluence. At this stage, uniform scratches were generated in the cell monolayers using a sterile 200 µl pipette tip. To ensure that wound closure reflected cell migration rather than proliferation, the cells were incubated with serum-free media containing 1 µg/mL mitomycin C. Wound healing progression was monitored by capturing images at 0, 12, 24, and 36 hours post-scratching under a microscope at 10 × magnification. The wound width at each time point was quantified using ImageJ software, and the wound closure rate was calculated based on the reduction in wound area over time.

### 2.14. Flow cytometry for apoptosis detection

Stable cell lines were plated in 6-well plates at 500,000 cells per well. Following the instructions of the Annexin V-FITC Apoptosis Detection Kit (C1062S, Beyotime, China), 300 µl of 1 × binding buffer and 5 µl of Annexin V-FITC were added, mixed thoroughly, and incubated in the dark for 10 minutes. Finally, 5 µl of PI and 200 µl of 1 × binding buffer were added 5 minutes before detection.

### 2.15. Statistical analysis

All statistical analyses were performed using Perl version 5.32.1 and R software version 4.4.1. Additionally, GraphPad Prism 10.0 (GraphPad Software Inc., USA) was used for statistical evaluation and visualization of data. A P-value<0.05 was considered statistically significant.

## 3. Results

### 3.1. Identification of DEGs in PCa

**Fig 2:**
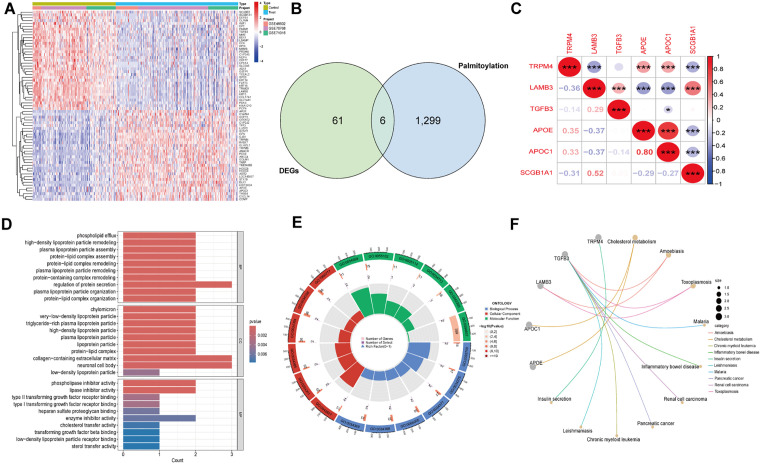
Differential analysis of integrated datasets, and GO/KEGG enrichment analysis. **(A)** Heatmap of the integrated dataset. **(B)** Venn diagrams of DEG and palmitoylation in the dataset. **(C)** Heatmap of palmitoylation-related genes. *, p < 0.05; **, p < 0.01; ***, p < 0.001. **(D-E)** The GO enrichment analysis. **(F)** Network Diagram of KEGG enrichment analysis. BP: biological process; CC: cellular component; MF: molecule function.

Using the “limma” R package, DEGs were identified from the GEO database (GSE46602, GSE70768, and GSE71016) in 197 PCa cases and 134 normal controls. Among them, the up-regulated genes with the most significant differential expression were HPN, TRPM4 and RAB17, and the down-regulated genes were GSTP1, TRIM29 and LAMB3 (S1 Fig). To find genes related to palmitoylation, we integrated genes obtained from the GeneCards database and relevant literature with the identified DEGs. The intersection of the two resulted in six overlapping DEGs, including three up-regulated and three down-regulated genes. These findings are visually represented in the heat map (Fig 2A-C).

### 3.2. Functional enrichment analysis

To elucidate the potential roles of the six palmitoylation-related genes in PCa, GO, KEGG, and GSEA analyses were performed using the “clusterProfiler” R package. The GO analysis showed that these genes were significantly enriched in biological processes related to lipid metabolism and protein modification (Fig 2D-E). Specifically, the biological processes included the organization of protein-lipid complexes, plasma lipoprotein particle organization, and regulation of protein secretion. Additionally, molecular functions such as cholesterol transfer activity, enzyme inhibitor activity, and heparan sulfate proteoglycan binding were closely associated with these genes. These results suggest that palmitoylation may play a significant role in lipid metabolism and signaling in prostate cancer. KEGG enrichment analysis indicated that the DEGs were primarily associated with cholesterol metabolism, amoebiasis, and toxoplasmosis (Fig 2F). The GSEA results showed that pathways enriched in the PCa group included ribosome, DNA adduct formation in chemical carcinogenesis, cytoskeleton in muscle cells, hypertrophic cardiomyopathy, and amoebiasis (S2 Fig).

### 3.3. Identification of optimal diagnostic gene biomarkers for PCa

**Fig 3:**
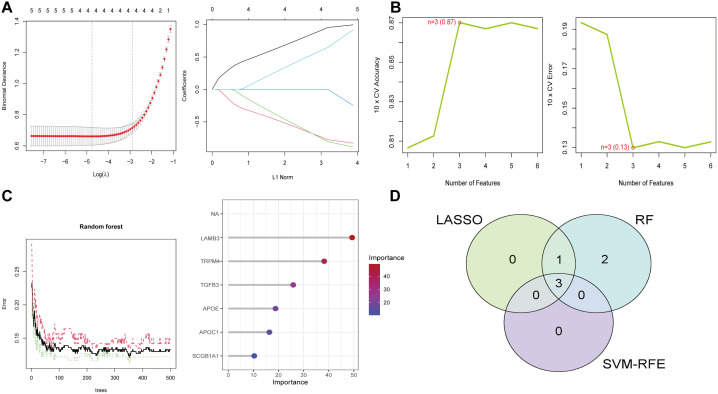
Screening of palmitoylation genes based on machine learning algorithms. **(A)** Results of Lasso regression analyses. **(B)** Results of SVM-RFE. **(C)** Results of RF modelling. **(D)** Venn diagrams showing crossover genes of LASSO, SVM-RFE and RF algorithms.

**Fig 4:**
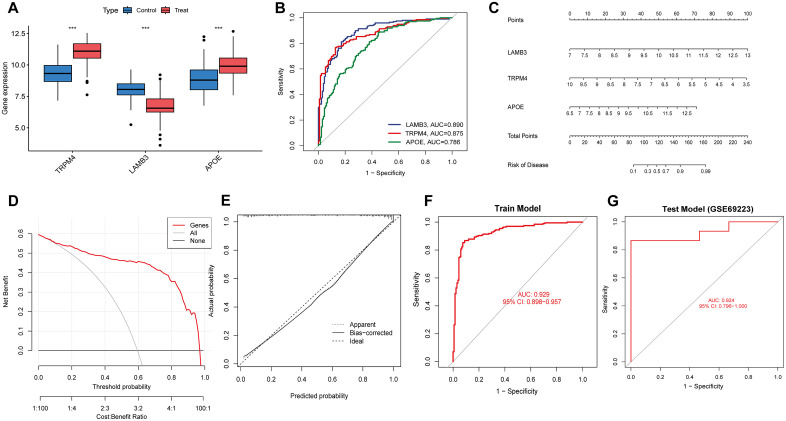
Diagnostic and validation analysis of model. **(A)** Expression analysis of model genes. **(B)** ROC verification of the model genes. **(C)** Diagnostic model genes in nomograms. **(D)** Calibration curve of the diagnostic model. **(E)** DCA plot of the diagnostic model. **(F)** ROC verification of the diagnostic models. **(G)** Validation of the diagnostic model in the test group GSE69223.

Given the differences between PCa patients and healthy individuals, our study aimed to identify the diagnostic potential of palmitoylation-related genes. Three machine learning methods such as LASSO, RF, and SVM-RFE were employed to effectively distinguish PCa from control samples (Fig 3A-D). LASSO logistic regression along with penalty parameter tuning and ten-fold cross-validation, identified four significant PCa-related features. Subsequently, SVM-RFE normalized an ideal set of three genes, while RF selected six core genes. The intersection of results from LASSO, RF, and SVM-RFE yielded three key genes: Transient Receptor Potential Melastatin 4 (TRPM4), LAMB3, and APOE, which were used for further evaluation. The expression of each core gene is shown in Fig 4A. A diagnostic model based on these three core genes in the integrated dataset achieved an AUC of 0.929 (Fig 4F). ROC curve validation for each key gene demonstrated diagnostic accuracy (Fig 4B). A nomogram was constructed based on the three core genes, showing that LAMB3 was much better at diagnosing the PCa model than the other variables. Decision Curve Analysis (DCA) was used to evaluate the clinical application of the model, which demonstrated favorable performance. Calibration curves further confirmed the model’s high diagnostic accuracy for PCa (Fig 4C-E). The diagnostic ability of our diagnostic model was also confirmed in the GSE69223 dataset (Fig 4G). Overall, these data highlight the precision and specificity of the logistic regression model in distinguishing PCa samples using independent marker genes.

**Fig 5:**
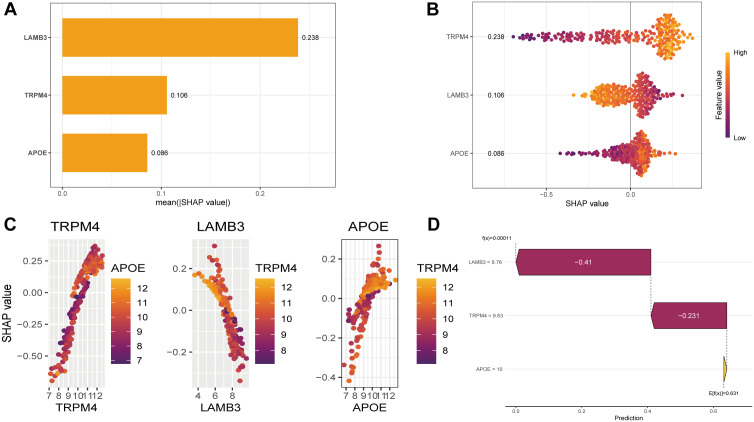
SHAP interpretable analyses on prognostic models screening for characterization genes that contribute the most. **(A)** Feature importance ranking bar chart. **(B)** Bee colony diagram. **(C)** Dependence Plot. **(D)** Waterfall diagram for localized explanations.

SHAP interpretability analysis revealed different functional contributions: LAMB3 (SHAP value = 0.238), TRMP4(SHAP value = 0.106), and APOE (SHAP value = 0.086), among which LAMB3 emerged as the most influential predictor (Fig 5A). At low expression levels, LAMB3 makes a significant contribution to peak prediction (Fig 5B), and has a strong interaction with TRMP4 (Fig 5C). Force-oriented analysis (Fig 5D) further demonstrated that LAMB3 (8.76, Δ = −0.41) and TRMP4 (9.63, Δ = −0.231) acted as the main negative modulators, driving the predicted value (f (x) = 0.00011) lower than the benchmark expectation (E[f (x)] = 0.631). Therefore, based on the results of the SHAP analysis, we chose LAMB3, which has the highest contribution to the model and the largest AUC among the model genes, as the object for further analysis.

### 3.4. Immune infiltration analysis

**Fig 6:**
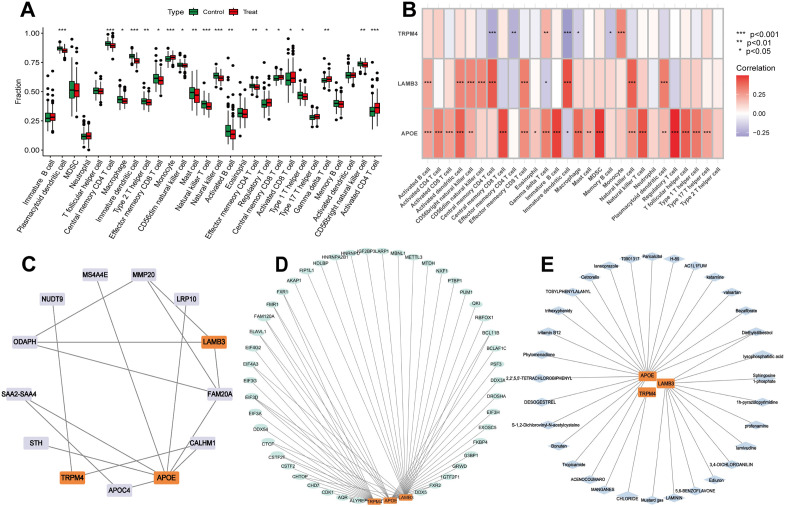
Immune infiltration analysis, and regulatory network of key genes. **(A)** Group comparison plot of 28 immune cells in different groups by ssGSEA. **(B)** Heatmap of correlation analysis between key genes and immune cell infiltration abundance by ssGSEA. **(C)** Protein-protein interaction networks (PPIs). **(D)** The mRNA-drugs interaction network. **(E)** The mRNA-RBP interaction network. RBP: RNA-binding protein.

Using ssGSEA, we assessed the correlation between the expression profiles of 28 immune cell types in PCa and normal groups. Based on the immune infiltration analysis and intergroup comparison boxplots, the abundance of 28 immune cell types was analyzed and visualized (Fig 6A). Significant differences (p < 0.05) were observed in the expression of 20 immune cell types, including plasmacytoid dendritic cells, central memory CD4 T cells, macrophages, immature dendritic cells, type 2 T helper cells, effector memory CD8 T cells, monocytes, CD56dim natural killer cells, mast cells, natural killer T cells, natural killer cells, activated B cells, effector memory CD4 T cells, regulatory T cells, central memory CD8 T cells, activated CD8 T cells, type 1 T helper cells, gamma delta T cells, CD56bright natural killer cells, and activated CD4 T cells. A correlation heatmap illustrated the relationship between the abundance of 28 statistically significant immune cell types (p < 0.05) and the expression of key genes (Fig 6B). APOE showed positive correlations with most immune cells, while TRPM4 exhibited negative correlations. While ssGSEA suggested correlations between APOE/LAMB3 and immune cell infiltration, bulk RNA-seq cannot distinguish cell-type-specific expression. SCGB1A1 and APOE may originate from stromal or immune cells rather than tumor epithelium. These findings require validation using spatial transcriptomics or flow cytometry.

### 3.5. mRNA-RBP, mRNA-TF, and mRNA-drug interaction networks

The STRING database analyses predicted protein interaction networks for key genes (Fig 6C). mRNA-RBP data from the ENCORI database were used to predict interactions between RBPs and key genes (Fig 6D). Using Cytoscape, an mRNA-RBP regulatory network was constructed, comprising 46 RBP molecules and 60 mRNA-RBP interactions. Subsequently, our enrichment analysis of the RBP results indicated that regulation of mRNA metabolic process, regulation of translation and RNA catabolic process are its main functions (S3 Fig). Analysis of DsigDB data identified potential drugs or compounds related to key genes (Fig 6E). An mRNA-drug regulatory network was created with Cytoscape, showing the five most important drugs or molecular compounds related to the core genes (Table 1)

**Table 1:** Enrichment analysis of drugs and target genes.

Drug	Count	pvalue	p.adjust	geneID
Diethylstilbestrol	2	0.000690127	0.005564857	LAMB3/APOE
Acenocoumarol	1	0.001983824	0.005564857	APOE
Lysophosphatidic acid	1	0.002441256	0.005564857	LAMB3
Tropicamide	1	0.002898549	0.005564857	APOE
Bonuten	1	0.003660393	0.005564857	APOE

### 3.6. Molecular docking

Based on drug regulatory network analysis, molecular docking was performed to investigate whether diethylstilbestrol targets LAMB3 and APOE to regulate palmitoylation modification (S4A–S4B Fig). The docking results showed that the drugs binding energies with APOE and LAMB3 protein were lower than −5 kcal·mol⁻1, suggesting the drug binds easily to the targets. Thus, LAMB3 and APOE may be potential targets for diethylstilbestrol in PCa treatment. Five conformations were output for each docking, and the conformation with the best binding energy was selected for visualization.

### 3.7. Cell transfection and validation

**Fig 7:**
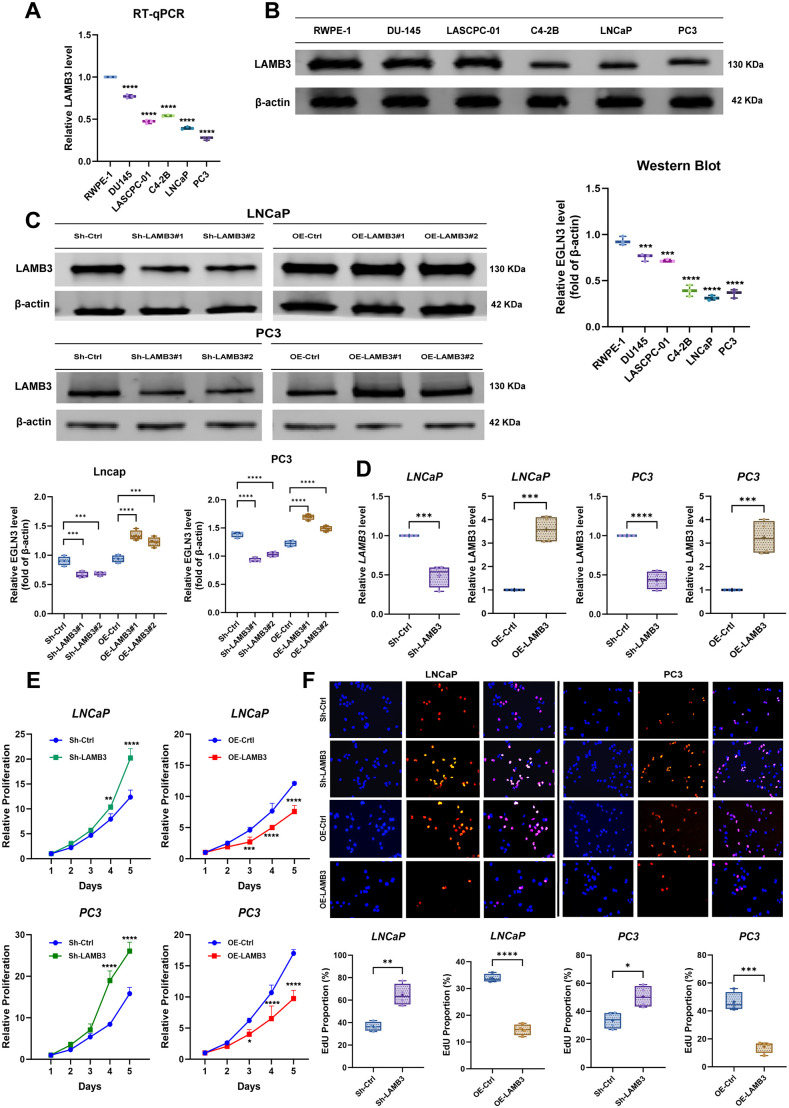
Establishment of stable cell lines and determination of proliferation ability. **(A)** Screening of prostate cancer cell lines. **(B,C)** Transfection efficiency and Gene expression level Verification, both mRNA and protein levels demonstrated statistically significant differences between the transfection cohorts and the control cohorts. **(D,E)** Both CCK-8 assays and EdU assays revealed that LAMB3 overexpression or knockdown significantly inhibited or enhanced the proliferative activity of LNCaP and PC3 cell lines. **(F)** The clone capacity of LNCaP and PC3 cells were inversely proportional to LAMB3 expression levels. *, p < 0.05; **, p < 0.01; ***, p < 0.001; ****, p < 0.0001.

We selected 5 human prostate cancer cell lines (DU145, LASCPC-01, C4-2B, LNCaP, PC3) and a normal prostate cell line (RWPE-1). RT-qPCR analysis and WB analysis showed that LAMB3 expression in LNCaP and PC3 cells were much lower than in other cell lines both at the mRNA level and protein level. Therefore, we selected LNCaP and PC3 cell lines to validate the phenotypic effects of the tumor suppressor gene LAMB3 in in vitro experiments. (Fig 7A-B)

In this study, PC3 and LNCaP cell lines were used to construct stable cell lines. After lentiviral transfection, stable cell lines—positive control (OE-Ctrl), overexpression (OE-LAMB3), negative control (Sh-Ctrl), and knockdown (Sh-LAMB3)—were obtained through puromycin screening for four weeks. Western blot and RT-qPCR were used to verify LAMB3 protein and mRNA expression, respectively, in the PC3 and LNCaP cell lines. The results confirmed the successful construction of stable cell lines (Fig 7C-D).

### 3.8. Validation of cell proliferation levels

#### 3.8.1. CCK-8 assay.

In the PC3 cell line, starting on Day 4, the knockdown group (Sh-LAMB3) demonstrated significantly higher proliferation levels compared to the control group (Sh-Ctrl), with a statistically significant difference. Similarly, in the overexpression group (OE-LAMB3), there was a noticeable reduction in proliferation levels starting from Day 3 when compared to its control group (OE-Ctrl). In the LNCaP cell line, the overexpression group (OE-LAMB3) showed a statistically significant decrease in proliferation levels starting on Day 3 compared to the OE-Ctrl group. Also, the knockdown group (Sh-LAMB3) showed a significant rise in proliferation levels by Day 4 compared to the Sh-Ctrl group (Fig 7E).

#### 3.8.2. EdU assay.

By merging the live cells labeled with the blue Hoechst dye and the proliferating cells labeled with the red Apize594 dye, the proliferation status of the cells in the field of view can be understood. Fig 7F shows representative images of different stably transfected cell lines of the PC3 and LNCaP cell lines. In the LNCaP cell line, we observed that the proliferation ratio of the knockdown group (Sh - LAMB3) cells is significantly lower than that of the control group (Sh – Crtl), while the proliferation ratio of the overexpression group (OE - LAMB3) cells is significantly higher than that of the control group (OE – Crtl), and these differences are statistically significant. Similarly, we also conducted an EdU assay to measure the proliferation of each stably transfected cell line in the PC3 cell line. The results are similar to those of the LNCaP cell line (Fig 7F).

### 3.9. Validation of cell cloning ability

**Fig 8:**
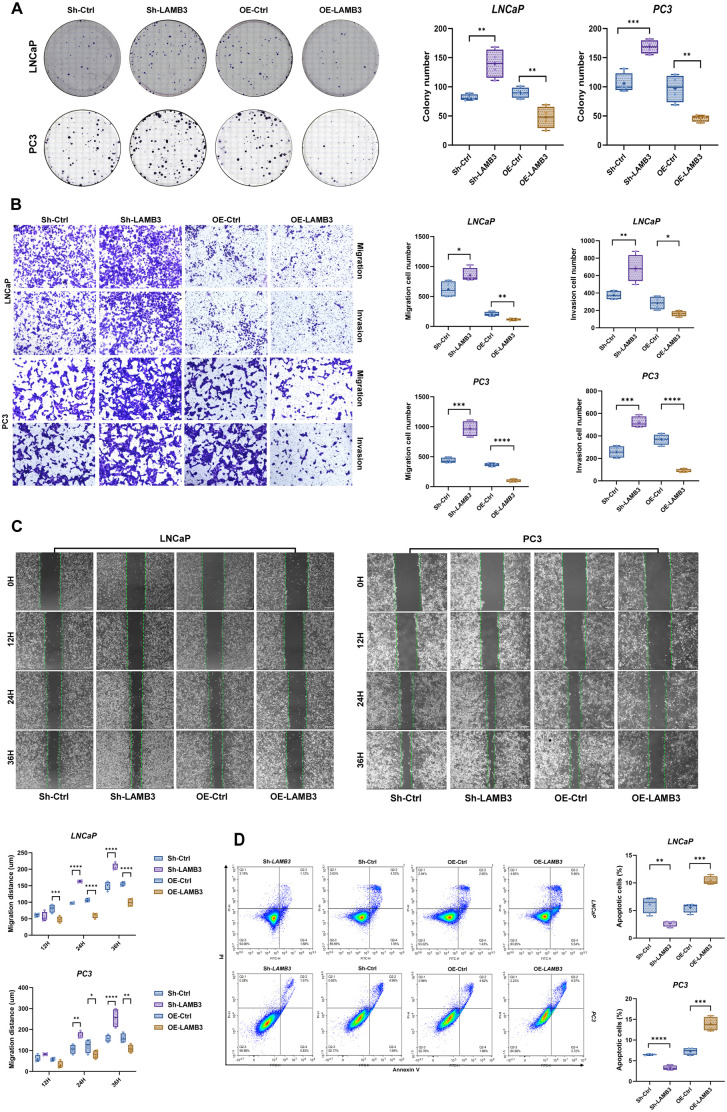
Determination of cell migration ability and apoptosis rate. **(A)** In the LNCaP cell line, the migratory capacity of cells in the overexpression cohort was significantly reduced, while no significant differences were observed in the knockdown cohort. In contrast, both overexpression and knockdown cohorts of PC3 cells exhibited statistically significant differences in migratory capacity compared to the control cohort. **(B)** In Both PC3 and LNCaP cell lines, the expression level of LAMB3 was positively correlated with the level of apoptosis. **(C)** In the LNCaP cell line, there is no significant differences were observed in the migratory capacity between knockdown cohort and control cohort. Conversely, the migratory capacity of cells in the overexpression cohort was significantly superior to that of the control cohort. *, p < 0.05; **, p < 0.01; ***, p < 0.001; ****, p < 0.0001.

The colony formation assay reflected the proliferative capacity and tumor cell population dependency. In both LNCaP and PC3 cell lines, the knockdown group (Sh-LAMB3) exhibited significantly enhanced colony formation ability compared to the control group (Sh-Ctrl), while the overexpression group (OE-LAMB3) showed significantly reduced colony formation ability compared to the control group (OE-Ctrl) (Fig 8A). These results were consistent with the CCK-8 and EdU assays.

### 3.10. Validation of cell migration and invasion abilities

#### 3.10.1. Transwell assay.

The “Transwell migration assay” accurately quantifies the ability of prostate cancer cells to respond to specific chemokines. The experimental results show that both in the LNCaP cell line and PC3 cell line, the migratory ability of cells in the knockdown group (Sh-LAMB3 group) is significantly enhanced compared with the control group (Sh-Ctrl). In contrast, the migratory ability of cells in the overexpression group (OE-LAMB3 group) is significantly reduced compared with the control group (OE-Ctrl). (Fig 8B).

Similarly, The “Transwell invasion assay” simulates the basement membrane penetration process in vivo and evaluates the ability of cells to secrete proteases to degrade the ECM, we further compared the invasive abilities among different cell groups by coating the upper chamber with Matrigel. The results demonstrated that both in the LNCaP cell line and PC3 cell line, the invasion capacity of the knockdown group (Sh-LAMB3) still showed significant changes compared to the control group (Sh-Ctrl), Conversely, the invasion ability of prostate cancer cells decreased significantly after LAMB3 overexpression. (Fig 8B).

#### 3.10.2. Wound healing assay.

Wound Healing Assay observe the dynamics of two-dimensional collective migration and repair, create scratches in monolayer cells, simulate wound healing or population migration, and track the collective movement pattern of cells. In LNCaP and PC3 cells, the knockdown group (Sh-LAMB3) showed significantly enhanced migration ability compared to the control group (Sh-Ctrl), while the overexpression group (OE-LAMB3) exhibited significantly reduced migration ability compared to the control group (OE-Ctrl). (Fig 8C).

### 3.11. Validation of apoptosis rate

In LNCaP and PC3 cells, the knockdown group (Sh-LAMB3) showed significantly lower early and late apoptosis rates compared to the control group (Sh-Ctrl), while the overexpression group (OE-LAMB3) exhibited significantly higher apoptosis rates compared to the control group (OE-Ctrl) (Fig 8D).

## 4. Discussion

Prostate cancer remains a leading cause of cancer-related mortality in men, posing significant challenges in oncology, particularly in addressing drug resistance, metastasis, and recurrence driven by genetic mutations [1–3]. Palmitoylation, a post-translational modification, plays a crucial role in processes such as cellular membrane localization, protein trafficking, and signal transduction. Increasing evidence suggests that palmitoylation significantly influences the initiation, progression, drug resistance, metastasis, and recurrence of prostate cancer [18–20]. For instance, Fiorentino et al. found that the fatty acid synthase (FASN), a cornerstone of protein palmitoylation, is overexpressed in prostate cancer and is associated with the palmitoylation of Wnt1 and the cytoplasmic stabilization of β-catenin, processes that may promote anti-apoptotic capabilities and proliferation in cancer cells [21,22]. In another study, De Piano et al. revealed that FASN regulates the adhesion and migration of prostate cancer cells by influencing the palmitoylation status of Rho family GTPases, particularly the atypical GTPase RhoU, highlighting FASN’s role in prostate cancer metastasis. These findings suggest that inhibiting FASN activity or its downstream palmitoylation events could effectively block prostate cancer progression, reduce drug resistance, and lower the risk of metastasis and recurrence [23]. Additionally, emerging evidence in recent years indicates that protein palmitoylation, a dynamic post-translational lipid modification, may regulate prostate cancer progression by modulating membrane localization and functional stability of oncoproteins, including extracellular matrix receptors and signaling kinases implicated in prostate cancer metastasis [24]. For instance, studies demonstrate that ZDHHC_5_ participates in modifying cancer stem-like properties through interaction with EZH2—a pathway previously observed in glioma stem cells that may share conserved mechanisms with prostate cancer [25]. Notably, altered palmitoylation of cell adhesion molecules and extracellular matrix regulators (e.g., laminin subunits) may facilitate tumor microenvironment remodeling, potentially explaining selective metastatic patterns in advanced prostate cancer [25]. However, the molecular targets, interactions, and diagnostic value of palmitoylation in prostate cancer remain unclear. Therefore, we employed bioinformatics approaches to explore the molecular mechanisms and diagnostic value of palmitoylation-related genes in prostate cancer pathogenesis and investigated potential therapeutic agents.

In this study, we merged three datasets from the GEO database and performed differential expression analysis between tumor and normal tissues to identify DEGs. By increasing the sample size through dataset integration, we enhanced the statistical significance and reliability of our results. Additionally, the diversity of sample data from different platforms and sources improved the model’s generalizability, making it applicable to a wide range of scenarios. By intersecting DEGs with known palmitoylation-related genes, we identified six overlapping genes, which were then input into three machine learning models, yielding three feature genes: TRPM4, LAMB3, and APOE. We constructed a diagnostic model using these feature genes. A diagnostic model and nomogram were established using logistic regression with these three biomarkers and their covariates, demonstrating high accuracy. ROC analysis was used to evaluate the individual diagnostic accuracy of the three biomarkers, revealing AUC values > 0.7 for all genes, indicating high diagnostic value.

TRPM4 is a non-selective cation channel activated by Ca2+. Upon activation, TRPM4 allows Na⁺ influx, leading to membrane depolarization and reduced driving force for Ca2+ entry through store-operated Ca²⁺ entry (SOCE) and other Ca2+ entry pathways. SOCE is associated with fundamental cellular processes such as gene expression, and its altered signaling contributes to cancer hallmarks, including reduced apoptosis, increased proliferation, and migration. TRPM4 also participates in focal adhesion protein co-localization in mouse embryonic fibroblasts and regulates focal adhesion turnover, which is critical for cell migration. In prostate cancer, TRPM4 is described as a cancer driver gene in androgen-independent prostate cancer. TRPM4 is responsible for Ca²⁺-activated non-selective (CAN) currents in prostate cancer, and TRPM4 knockdown reduces large Na⁺ currents following Ca2+ activation. TRPM4 contributes to the migration and invasion of the PC3 prostate cancer cell line and alters epithelial-mesenchymal transition (EMT), a crucial process for cancer cell migration and invasion. TRPM4 downregulation induces changes in E-cadherin and N-cadherin expression levels and reduces the expression of Snail1, a known EMT marker transcription factor [26].

APOE, a member of the lipid-binding apolipoprotein family, is a major systemic transporter of cholesterol and triglycerides. The role of APOE in prostate cancer is multifaceted, with studies showing its close association with tumor progression and prognosis. First, APOE expression in prostate cancer correlates with tumor aggressiveness and hormone independence. Venanzoni et al. found that APOE mRNA is highly expressed in the highly tumorigenic PC-3 cell line. Immunohistochemical analysis revealed that APOE protein expression positively correlates with Gleason scores, suggesting that elevated APOE expression in prostate cancer cells may be associated with high invasiveness. Further studies have explored the impact of APOE gene polymorphisms on prostate cancer [27]. Ifere et al. revealed a link between APOE gene variants and aggressive prostate cancer behavior. They found that non-invasive cell lines carry ApoE ε3/ε3 or ε3/ε4 alleles, while invasive cell lines carry APOE ε2/ε4 alleles, indicating that APOE variants are associated with intracellular cholesterol imbalance and tumor aggressiveness [28]. Finally, Bancaro et al. provided new insights into APOE’s role in prostate cancer. They discovered that APOE secreted by prostate tumor cells induces the senescence of TREM2 ⁺ immunosuppressive neutrophils, which are associated with poor prognosis in prostate cancer patients. Eliminating these senescent neutrophils using histone deacetylase (HDAC) inhibitors can improve cancer treatment outcomes [29].

LAMB3 is a key component of the laminin 5 complex, responsible for cell adhesion in the extracellular matrix and regulating cell proliferation, migration, and cycle in various diseases. The development and metastasis of prostate cancer are closely linked to the abnormal activation of the PTEN/AKT/mTOR pathway. Studies have shown that SAG, an E3 ligase, activates the PI3K/AKT/mTOR signaling axis by promoting the ubiquitination and degradation of PHLPP1 and DEPTOR. In PTEN-deficient prostate cancer mouse models, SAG deletion suppresses the abnormal activation of PI3K/AKT/mTOR signaling. Additionally, defects in laminin 5 expression are commonly observed in prostate cancer [30]. Calaluce et al. demonstrated that H3 B subtype cells expressing LAMB3 cannot translate the protein or assemble laminin 5, promoting prostate cancer proliferation and invasion. In contrast, the H3 A subtype increases LAMB3 expression but also fails to assemble laminin 5, further driving prostate cancer progression. These findings highlight the critical role of LAMB3 in prostate cancer signaling [31].

Furthermore, our study explored the relationship between palmitoylation-related genes and immune cell infiltration. Using ssGSEA analysis, we identified significant differences in the expression profiles of 28 immune cell types between prostate cancer and normal tissues, which may be related to the regulation of the tumor microenvironment. Among these, myeloid dendritic cells (mDCs) play a key role in initiating and regulating adaptive anti-tumor immune responses. Immature dendritic cells may share similar functions with mDCs, presenting tumor antigens to T cells. LAMB3, an extracellular matrix protein, may be involved in dendritic cell migration and antigen presentation. T cell subsets, including central memory CD4 T cells and effector memory CD8 T cells, exhibit heterogeneity in prostate cancer. APOE may influence T cell function by modulating lipid metabolism and immune regulation, while LAMB3 may participate in tumor immune responses by affecting T cell migration and localization. These findings provide new insights into the immune microenvironment of prostate cancer and may aid in developing novel immunotherapeutic strategies.

Finally, we overexpressed and knocked down LAMB3 in prostate cancer cells using lentiviral plasmid transfection, followed by functional experiments to validate LAMB3 as a tumor suppressor gene that inhibits prostate cancer progression. In terms of proliferation, invasion, migration and apoptosis rates, LAMB3 expression levels were inversely correlated with prostate cancer progression, showing statistically significant differences.

This study used bulk RNA-seq data from adjacent normal tissues, which may include stromal or inflammatory cells. While we excluded samples with diagnosed benign prostatic hyperplasia (BPH), age-related pre-cancerous changes in normal tissues could theoretically influence DEG identification. Future single-cell RNA-seq studies are warranted to resolve cell-type-specific palmitoylation signatures. Additionally, although we employed multiple algorithms, including RF, LASSO regression, SVM-RFE, and logistic regression, to identify and validate candidate biomarkers, differences in results across algorithms suggest the need for model validation and optimization on broader datasets to ensure accuracy and consistency. Furthermore, while this study primarily used statistical and machine learning techniques to screen potential biomarkers, the biological functions and mechanisms of these candidate genes require further experimental validation. Subsequent work should involve in vitro and in vivo experiments to elucidate the specific roles of these genes in prostate cancer initiation and progression. Meanwhile, the putative immune correlations (e.g., APOE with macrophages) should be interpreted cautiously, as bulk RNA-seq lacks resolution to disentangle tumor-intrinsic vs. microenvironmental contributions. Our immune infiltration analysis did not adjust for multiple comparisons, which increases the risk of Type I errors. Findings related to specific immune cell types require independent validation in larger cohorts before definitive conclusions can be drawn. Future work will: Map site-specific palmitoylation of LAMB3/APOE using mass spectrometry; Validate the diagnostic model in prospectively collected clinical samples; Test therapeutic modulation of palmitoylation using diethylstilbestrol.

## 5. Conclusion

In conclusion, our study has identified novel palmitoylation-related molecular biomarkers and established a promising diagnostic model for prostate cancer. These discoveries have not only enhanced the understanding of molecular mechanisms underlying palmitoylated genes in prostate cancer pathogenesis, but also provided a scientific foundation for developing innovative therapeutic strategies. With the advancement of precision medicine, we substantiate that these palmitoylation-regulated genes will emerge as critical therapeutic targets in personalized treatment regimens for prostate cancer

## Supporting information

S1 Fig Integration of Differentially Expressed Genes. Combine the expression levels of genes with significant differential expression in the dataset. (A) HPN; (B) TRPM4; (C) RAB17; (D) GSTP1; (E) TRIM29; (F) LAMB3.
(TIF)

S2 Fig GSEA Identifies Five Significantly Enriched Pathways Including Ribosome and Cardiomyopathy. GSEA of the merged dataset. (A) GSEA was used to provide five biological function maps for the genome. (B-F) GSEA results showed that the genome was significantly enriched in Ribosome pathway (B), Cytoskeleton in muscle cells pathway (C), Amoebiasis pathway (D), Chemical carcinogenesis – DNA adducts pathway (E), Hypertrophic cardiomyopathy pathway (F).
(TIF)

S3 Fig Enrichment analysis of mRNA-RBP. (TIF)

S4 Fig Optimal Drug-Target Docking Visualization. Visual representation of the optimal combination of drug-target gene molecule docking. (A)Diethylstilbestrol and APOE; (B) Diethylstilbestrol and LAMB3.
(TIF)

S1 Table List of palmitoylation-related genes. (CSV)

## References

[1] Sung H, Ferlay J, Siegel RL, Laversanne M, Soerjomataram I, Jemal A, et al. Global Cancer Statistics 2020: GLOBOCAN Estimates of Incidence and Mortality Worldwide for 36 Cancers in 185 Countries. CA Cancer J Clin. 2021;71(3):209–49. doi: 10.3322/caac.21660 33538338

[2] Bray F, Laversanne M, Sung H, Ferlay J, Siegel RL, Soerjomataram I, et al. Global cancer statistics 2022: GLOBOCAN estimates of incidence and mortality worldwide for 36 cancers in 185 countries. CA Cancer J Clin. 2024;74(3):229–63. doi: 10.3322/caac.21834 38572751

[3] Lowrance W, Dreicer R, Jarrard DF, Scarpato KR, Kim SK, Kirkby E, et al. Updates to Advanced Prostate Cancer: AUA/SUO Guideline (2023). J Urol. 2023;209(6):1082–90. doi: 10.1097/JU.0000000000003452 37096583

[4] Schröder FH, Hugosson J, Roobol MJ, Tammela TLJ, Ciatto S, Nelen V, et al. Screening and prostate-cancer mortality in a randomized European study. N Engl J Med. 2009;360(13):1320–8. doi: 10.1056/NEJMoa0810084 19297566

[5] Gillessen S, Bossi A, Davis ID, de Bono J, Fizazi K, James ND, et al. Management of patients with advanced prostate cancer-metastatic and/or castration-resistant prostate cancer: Report of the Advanced Prostate Cancer Consensus Conference (APCCC) 2022. Eur J Cancer. 2023;185:178–215. doi: 10.1016/j.ejca.2023.02.018 37003085

[6] Agarwal N, McGregor B, Maughan BL, Dorff TB, Kelly W, Fang B, et al. Cabozantinib in combination with atezolizumab in patients with metastatic castration-resistant prostate cancer: results from an expansion cohort of a multicentre, open-label, phase 1b trial (COSMIC-021). Lancet Oncol. 2022;23(7):899–909. doi: 10.1016/S1470-2045(22)00278-9 35690072

[7] Fizazi K, Tran N, Fein L, Matsubara N, Rodriguez-Antolin A, Alekseev BY, et al. Abiraterone plus Prednisone in Metastatic, Castration-Sensitive Prostate Cancer. N Engl J Med. 2017;377(4):352–60. doi: 10.1056/NEJMoa1704174 28578607

[8] Shang S, Liu J, Hua F. Protein acylation: mechanisms, biological functions and therapeutic targets. Signal Transduct Target Ther. 2022;7(1):396. doi: 10.1038/s41392-022-01245-y 36577755 PMC9797573

[9] S Mesquita F, Abrami L, Linder ME, Bamji SX, Dickinson BC, van der Goot FG. Mechanisms and functions of protein S-acylation. Nat Rev Mol Cell Biol. 2024;25(6):488–509. doi: 10.1038/s41580-024-00700-8 38355760 PMC12010433

[10] Das T, Yount JS, Hang HC. Protein S-palmitoylation in immunity. Open Biol. 2021;11(3):200411. doi: 10.1098/rsob.200411 33653086 PMC8061762

[11] Ko P, Dixon SJ. Protein palmitoylation and cancer. EMBO Reports. 2018;19(10). doi: 10.15252/embr.201846666 PMC6172454 30232163

[12] Zhou B, Hao Q, Liang Y, Kong E. Protein palmitoylation in cancer: molecular functions and therapeutic potential. Mol Oncol. 2023;17(1):3–26. doi: 10.1002/1878-0261.13308 36018061 PMC9812842

[13] Horita H, Law A, Middleton K. Utilizing Optimized Tools to Investigate PTM Crosstalk: Identifying Potential PTM Crosstalk of Acetylated Mitochondrial Proteins. Proteomes. 2018;6(2):24. doi: 10.3390/proteomes6020024 29786648 PMC6027404

[14] Yang J, Xu J, Wang W, Zhang B, Yu X, Shi S. Epigenetic regulation in the tumor microenvironment: molecular mechanisms and therapeutic targets. Signal Transduct Target Ther. 2023;8(1):210. doi: 10.1038/s41392-023-01480-x 37217462 PMC10203321

[15] Vlajnic T, Bubendorf L. Molecular pathology of prostate cancer: a practical approach. Pathology. 2021;53(1):36–43. doi: 10.1016/j.pathol.2020.10.003 33234230

[16] Burska D, Stiburek L, Krizova J, Vanisova M, Martinek V, Sladkova J, et al. Homozygous missense mutation in UQCRC2 associated with severe encephalomyopathy, mitochondrial complex III assembly defect and activation of mitochondrial protein quality control. Biochim Biophys Acta Mol Basis Dis. 2021;1867(8):166147. doi: 10.1016/j.bbadis.2021.166147 33865955

[17] Crofts AR, Hong S, Wilson C, Burton R, Victoria D, Harrison C, et al. The mechanism of ubihydroquinone oxidation at the Qo-site of the cytochrome bc1 complex. Biochim Biophys Acta. 2013;1827(11–12):1362–77. doi: 10.1016/j.bbabio.2013.01.009 23396004 PMC3995752

[18] Gottlieb CD, Linder ME. Structure and function of DHHC protein S-acyltransferases. Biochem Soc Trans. 2017;45(4):923–8. doi: 10.1042/BST20160304 28630137

[19] Qu M, Zhou X, Wang X, Li H. Lipid-induced S-palmitoylation as a Vital Regulator of Cell Signaling and Disease Development. Int J Biol Sci. 2021;17(15):4223–37. doi: 10.7150/ijbs.64046 34803494 PMC8579454

[20] Fukata Y, Murakami T, Yokoi N, Fukata M. Local Palmitoylation Cycles and Specialized Membrane Domain Organization. Curr Top Membr. 2016;77:97–141. doi: 10.1016/bs.ctm.2015.10.003 26781831

[21] Pepinsky RB, Zeng C, Wen D, Rayhorn P, Baker DP, Williams KP, et al. Identification of a palmitic acid-modified form of human Sonic hedgehog. J Biol Chem. 1998;273(22):14037–45. doi: 10.1074/jbc.273.22.14037 9593755

[22] Gao X, Hannoush RN. Single-cell imaging of Wnt palmitoylation by the acyltransferase porcupine. Nat Chem Biol. 2014;10(1):61–8. doi: 10.1038/nchembio.1392 24292069

[23] De Piano M, Manuelli V, Zadra G, Loda M, Muir G, Chandra A, et al. Exploring a role for fatty acid synthase in prostate cancer cell migration. Small GTPases. 2021;12(4):265–72. doi: 10.1080/21541248.2020.1826781 33043786 PMC8205051

[24] Wlodarczyk J, Bhattacharyya R, Dore K, Ho GPH, Martin DDO, Mejias R, et al. Altered Protein Palmitoylation as Disease Mechanism in Neurodegenerative Disorders. J Neurosci. 2024;44(40):e1225242024. doi: 10.1523/JNEUROSCI.1225-24.2024 39358031 PMC11450541

[25] Fan X, Gong M, Yu H, Yang H, Wang S, Wang R. Propofol enhances stem-like properties of glioma via GABAAR-dependent Src modulation of ZDHHC5-EZH2 palmitoylation mechanism. Stem Cell Res Ther. 2022;13(1):398. doi: 10.1186/s13287-022-03087-5 35927718 PMC9351178

[26] Launay P, Fleig A, Perraud A-L, Scharenberg AM, Penner R, Kinet J-P. TRPM4 Is a Ca2+-Activated Nonselective Cation Channel Mediating Cell Membrane Depolarization. Cell. 2002;109(3):397–407. doi: 10.1016/s0092-8674(02)00719-5 12015988

[27] Venanzoni MC, Giunta S, Muraro GB, Storari L, Crescini C, Mazzucchelli R, et al. Apolipoprotein E expression in localized prostate cancers. Int J Oncol. 2003;22(4):779–86. 12632068

[28] Ifere GO, Desmond R, Demark-Wahnefried W, Nagy TR. Apolipoprotein E gene polymorphism influences aggressive behavior in prostate cancer cells by deregulating cholesterol homeostasis. Int J Oncol. 2013;43(4):1002–10. doi: 10.3892/ijo.2013.2057 23934233 PMC3829771

[29] Bancaro N, Calì B, Troiani M, Elia AR, Arzola RA, Attanasio G, et al. Apolipoprotein E induces pathogenic senescent-like myeloid cells in prostate cancer. Cancer Cell. 2023;41(3):602-619.e11. doi: 10.1016/j.ccell.2023.02.004 36868226

[30] Tan M, Xu J, Siddiqui J, Feng F, Sun Y. Depletion of SAG/RBX2 E3 ubiquitin ligase suppresses prostate tumorigenesis via inactivation of the PI3K/AKT/mTOR axis. Mol Cancer. 2016;15(1):81. doi: 10.1186/s12943-016-0567-6 27955654 PMC5153812

[31] Calaluce R, Bearss DJ, Barrera J, Zhao Y, Han H, Beck SK, et al. Laminin-5 β3A Expression in LNCaP Human Prostate Carcinoma Cells Increases Cell Migration and Tumorigenicity. Neoplasia. 2004;6(5):468–79. doi: 10.1593/neo.03499 15548355 PMC1531651

