# Supplementary figures and images for: Identification and validation of palmitoylation-related signature genes based on machine learning for prostate cancer
Source: PLoS One. 2025 Dec 4;20(12):e0338407. doi: 10.1371/journal.pone.0338407 (PMC12677499; doi:10.1371/journal.pone.0338407)

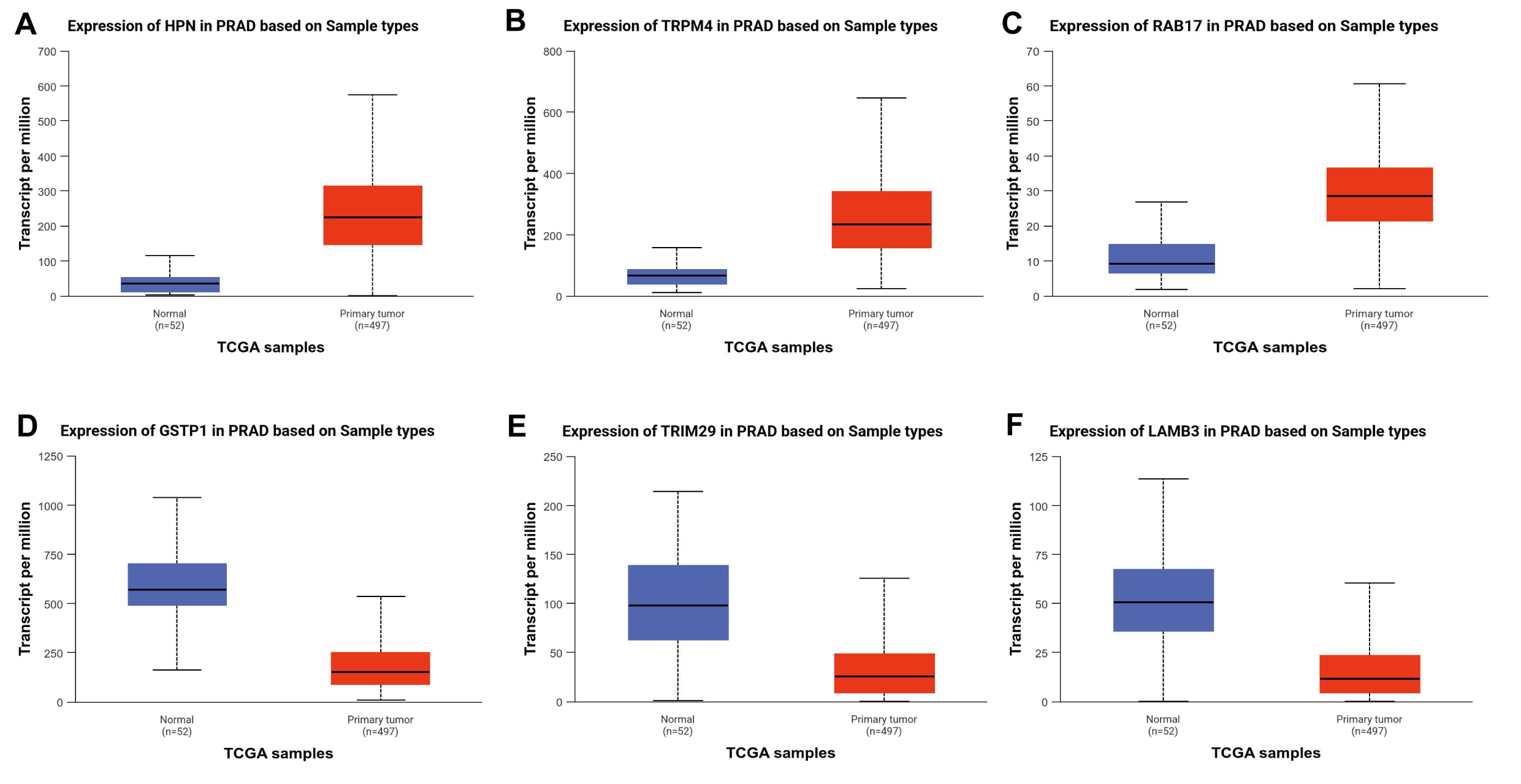

Supplement: S1 Fig — Combine the expression levels of genes with significant differential expression in the dataset. (A) HPN; (B) TRPM4; (C) RAB17; (D) GSTP1; (E) TRIM29; (F) LAMB3. (TIF) [file pone.0338407.s001.tif]

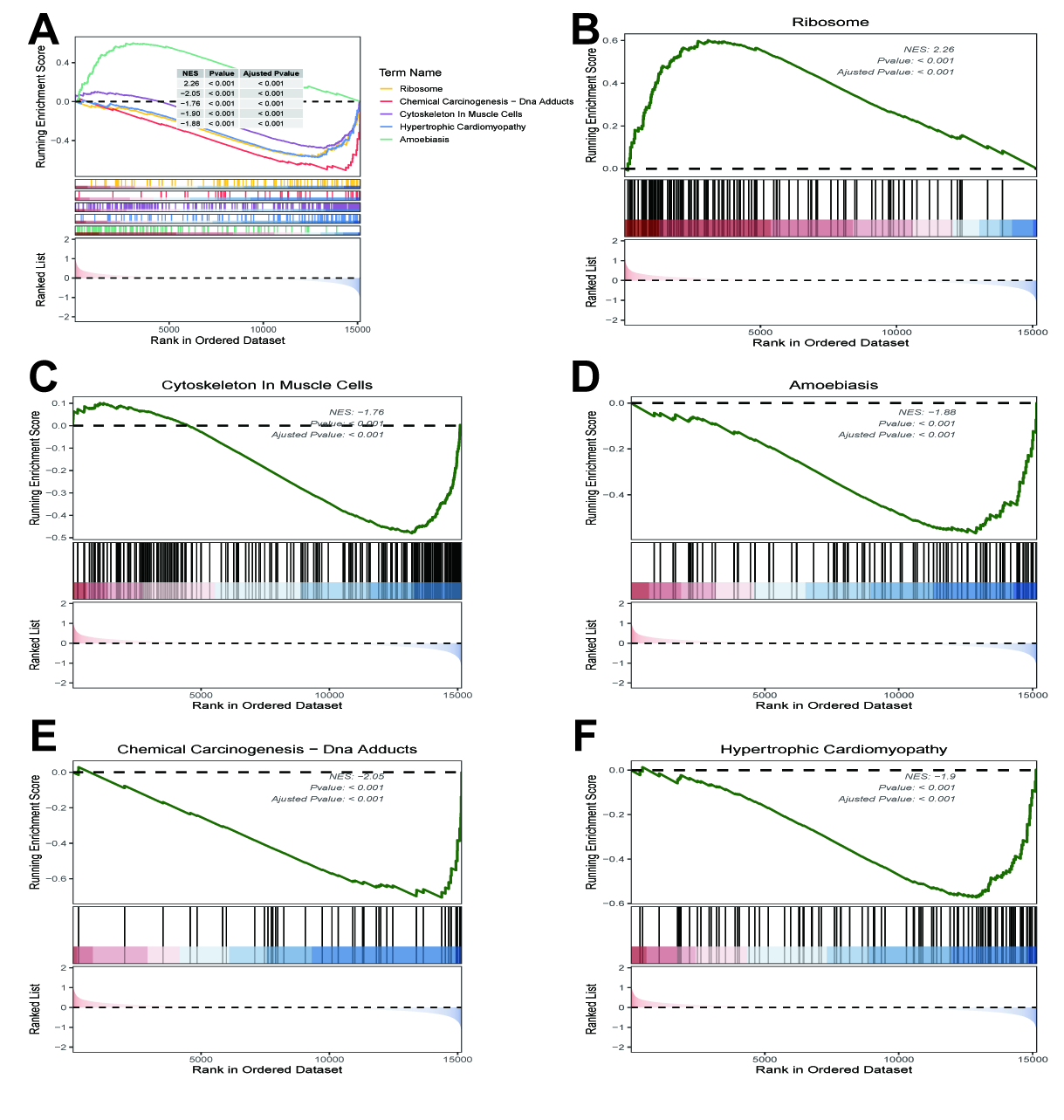

Supplement: S2 Fig — GSEA of the merged dataset. (A) GSEA was used to provide five biological function maps for the genome. (B-F) GSEA results showed that the genome was significantly enriched in Ribosome pathway (B), Cytoskeleton in muscle cells pathway (C), Amoebiasis pathway (D), Chemical carcinogenesis – DNA adducts pathway (E), Hypertrophic cardiomyopathy pathway (F). (TIF) [file pone.0338407.s002.tif]

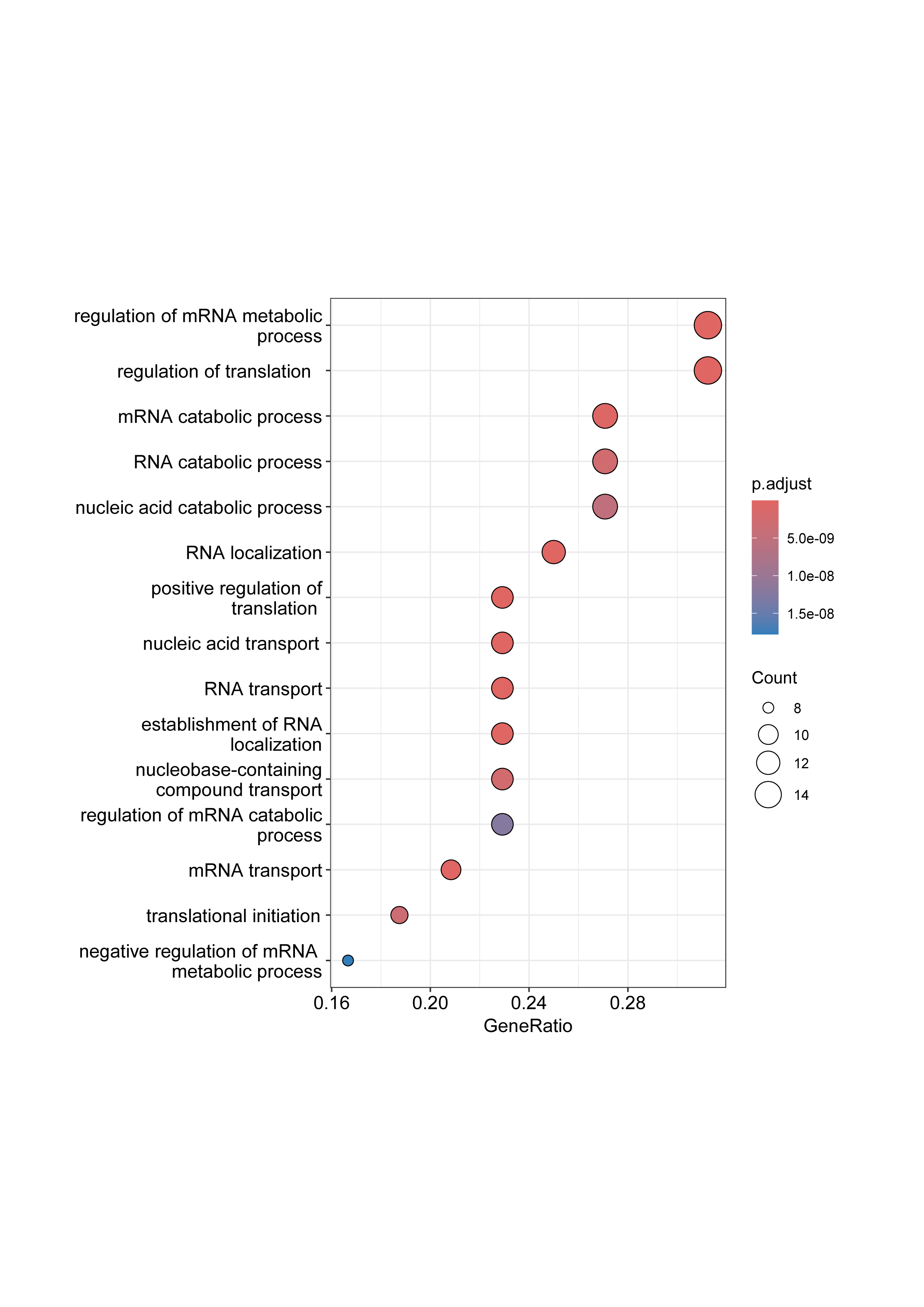

Supplement: S3 Fig — (TIF) [file pone.0338407.s003.tif]

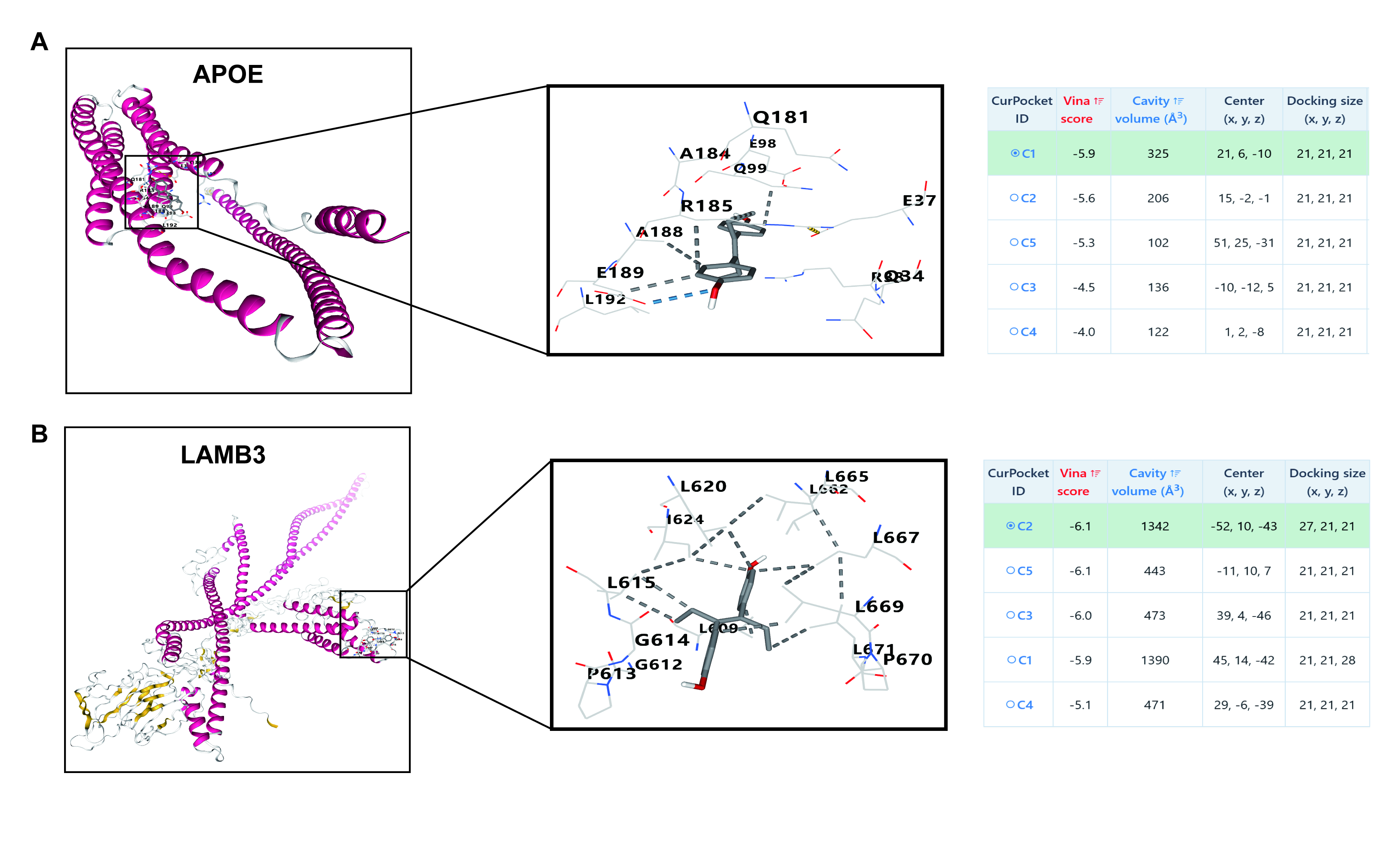

Supplement: S4 Fig — Visual representation of the optimal combination of drug-target gene molecule docking. (A)Diethylstilbestrol and APOE; (B) Diethylstilbestrol and LAMB3. (TIF) [file pone.0338407.s004.tif]
